# Where age doesn’t matter: no relative age effects in esports at the professional level

**DOI:** 10.3389/fspor.2025.1699838

**Published:** 2025-11-10

**Authors:** Aron Laxdal, Martin Kjeøen Erikstad

**Affiliations:** Department of Sport Science and Physical Education, University of Agder, Kristiansand, Norway

**Keywords:** electronic sports, Galatea Effect, gaming, Pygmalion Effect, Matthew Effect

## Abstract

Relative age effects (i.e., a systematic bias favouring individuals born earlier in the selection year) have been found in many traditional sports, especially among younger athletes in sports where physical maturation (e.g., increased size and strength) is advantageous. The aim of this study was to explore relative age effects in professional esports, which differ from traditional sports in some key areas. The birth months of 15,734 e-sport athletes playing ten different games were collected from Liquipedia and analyzed using chi-square tests. The analyses showed no practically meaningful relative age effects among the athletes, indicating that being relatively older does not yield advantages in gameplay. The same results applied across the various age groups as well as the different game types. This lack of relative age effects can likely be attributed to the cognitive and skill-based nature of esports, as well as the flexible and online nature of competitive environments in this domain

## Introduction

In recent years, esports have increasingly mirrored traditional sports in terms of competitiveness, high-stakes tournaments, and the emergence of high-profile athletes. This rapid growth has captivated a global audience and transformed competitive gaming into a multimillion-dollar industry (1). As esports gain recognition as a legitimate competitive domain, researchers have begun to explore the factors that contribute to success. One such area of interest is the relative age effect, a phenomenon extensively studied in traditional sports but less understood in esports (2).

The relative age effect refers to a systematic bias favoring individuals born earlier in the selection year. First identified in sports (3), it has been widely documented in youth and professional contexts. Relative age effects have been observed across many sports (4–6) and in both male (7) and female athletes (8), though they are usually more pronounced in males [e.g., (9)]. Generally, relative age effects are strongest in competitive youth contexts (2), but they can also appear at the professional level, although with more inconsistent results (5, 10).

The prevalence of relative age effects varies depending on the type of sport. Technical or cognitive domains, where skill and strategy outweigh physical maturity, often show weaker or less consistent effects (11, 12). In chess, for example, relative age effects tend to favor older youth, particularly females, while findings for males are mixed, with some studies reporting reversed effects that may be influenced by cultural or external factors (13, 14). Beyond sports, relative age effects have also been linked to peer relationships and mental health (15), ADHD diagnoses (16), health outcomes (17), and academic achievement (18), highlighting their broader developmental importance.

Several mechanisms have been proposed to explain why relative age effects occur. Hancock et al. (19) describe three key processes. The Matthew Effect suggests that early advantages create cumulative benefits over time. The Pygmalion Effect highlights the role of external expectations, where coaches and peers assume earlier-born athletes are more capable, which then influences performance. The Galatea Effect emphasizes self-belief, where athletes who see themselves as capable are more likely to put forth greater effort and achieve higher levels of success. Together, these processes create reinforcing cycles that advantage those born earlier in the selection year.

In traditional sports, researchers have also suggested ways to reduce relative age effects. These include varying cut-off dates for age categories (20), raising awareness among coaches (21), and placing more emphasis on technical skills rather than growth-related physical attributes (19). However, several features of esports could reduce relative age effects. Players are typically grouped by skill, not age, and physical size or strength plays little role in success. Progress often comes through open participation and ranked ladders/tournaments, with less early exclusion based on factors related to physical maturity. Indeed, the pathway to professional level in traditional sports often includes early formal selection into specialized clubs or academies to nurture the talent of the most promising athletes, where esports typically don't have formal systems of early recruitment or talent pipelines, and athletes can in theory become professionals at a very young age (22). Because players can continue competing and developing in an apprenticeship model without passing early selection gates [see also (23)], dropout pressures may be lower, which could weaken relative age effects. In addition, competition often takes place in online environments, where limited face-to-face interaction reduces the social pressures and expectations that might otherwise exaggerate perceived differences between players. That said, not all games are the same, and teams and academies introduce tryouts, and scouting can create selection pressures. Overall, these characteristics suggest that relative age effects may be limited in esports.

However, existing evidence complicates this expectation. Jakobsson et al. (24) examined relative age effects across sports in Sweden and reported that younger esports players under the age of 15 showed evidence of relative age effects, while players aged 21–39 showed an inverse effect. Their sample, however, was highly heterogeneous, including all registered esports athletes (*n* = 47,030) rather than focusing specifically on professionals. These findings suggest that esports may not fit neatly into the patterns observed in either physical or cognitive domains, but it remains unclear whether they also exist at the highest competitive level. To address this gap, the objective of the present study was to investigate whether relative age effects are present among professional esports, and to examine variation across game types and age cohorts.

## Method

### Participants and procedure

Liquipedia (www.liquipedia.net) is a community-based encyclopedia specialized in esports. Among the information posted on the website are the birthdays of current and former professional esports athletes. To get a profile in the Liquipedia database, an athlete has to exceed a certain threshold of notability. The criteria vary depending on the game, but are usually determined by personal achievements (i.e., placements and earnings in prestigious tournaments) and which roster or organization the player belongs to (see examples of notability criteria for Counter Strike (25) and Valorant (26). We collected the birth month of every registered player in the following games: Counter Strike, League of Legends, Call of Duty, Dota 2, StarCraft II, Valorant, Overwatch, Apex Legends, FIFA, and Fortnite; a total of 16,320 players. We chose these specific games as they were the 10 PC games on Liquipedia with the most complete athlete records. Phone-based games were not of interest.

Some players (*n* = 550) had reached notability in more than one of the abovementioned games. When all duplicates had been removed (matching on name and birthdate), we ended up with 15,734 unique players (M_age_ = 26.40; SD = 5.24). We did not analyze sex because professional esports typically are not sex segregated. Some of the athletes within the database have stopped playing actively and have moved on to other roles within the esports community, but we did not exclude them as we were interested in potential generational differences. To ensure the validity of the data, we manually verified the birthdays of 250 randomly selected players (25 from each game), finding no anomalies.

The players were categorized into quartiles depending on their birth month. All players born between January and March were classified into quartile one (Q1), players born between April and June were classified into quartile two (Q2), all players born between July and September were classified into quartile three (Q3), and all players born between October and December were classified into quartile four (Q4). The players’ age was calculated using their birthyear (2024—birthyear), and they were subsequently divided into three approximately equal age categories based on rank-order categorization (<24 years old [M_age_ = 21.04; SD = 1.71], 24-27 years old [M_age_ = 25.43; SD = 1.11], and >27 years old [M_age_ = 32.02; SD = 3.83). The various games included were classified as either real-time strategy games (Star Craft II), first person shooter games (Counter Strike, Valorant, Overwatch, Call of Duty, Apex Legends, and Fortnite), multiplayer online battle arena games (League of Legends and Dota 2), or traditional sports games (FIFA), in accordance with commonly used classifications [see e.g., (1)].

### Statistical analysis

A Chi-square goodness of fit test was used to compare differences between the observed and expected prevalence of births within each quartile. In line with the tradition within the literature [see (27)], we assumed an equal distribution of 25% within each quartile. However, it should be noted that births within a country may not be evenly distributed across the months, possibly due to factors such as weather patterns, cultural practices and holiday celebrations. For instance, studies using national population statistics from UK and Canada has found higher number of births in April-August (27). Nevertheless, an expected equal distribution was deemed most accurate for the present study given the global sample investigated. After the overall test, we conducted two-sided one-sample binomial test comparing each quartile proportion to .25, applying a Bonferroni correction for four comparisons.

Contingency tables (i.e., chi-square tests of independence) were used to compare the distribution of births across age groupings (four quartiles −1*three age groups −1 = 6 df) and game types (four quartiles −1 * five game types −1 = 12 df). Seeing as our sample is as large as it is, and Chi-square tests being sensitive to large sample sizes, we were not solely interested in finding statistically significant results (i.e., *p* < .05), but rather practically significant results. Based on previous results from professional sports that have mostly reported small effect sizes [e.g. (8, 27, 28),], we set small as our smallest effect size of interest (Cohen, 1988). Because different analyses use different effect sizes and benchmarks, we report the standard thresholds for each. The effect size for the goodness-of-fit test is Cohen's w and the benchmarks are .10 (small), .30 (medium), and .50 (large). Cramer's V is the effect size for chi-square test of independence, and the benchmarks differ depending on the contingency table. The benchmarks for the 4 × 3 table are .071 (small), .212 (medium), and .354 (large), while the benchmarks for the 4 × 5 table are .058 (small), .173 (medium), and .289 (large; Cohen, 1988). All statistical analyses were done in SPSS (Version 29.0).

## Results

**Figure 1:**
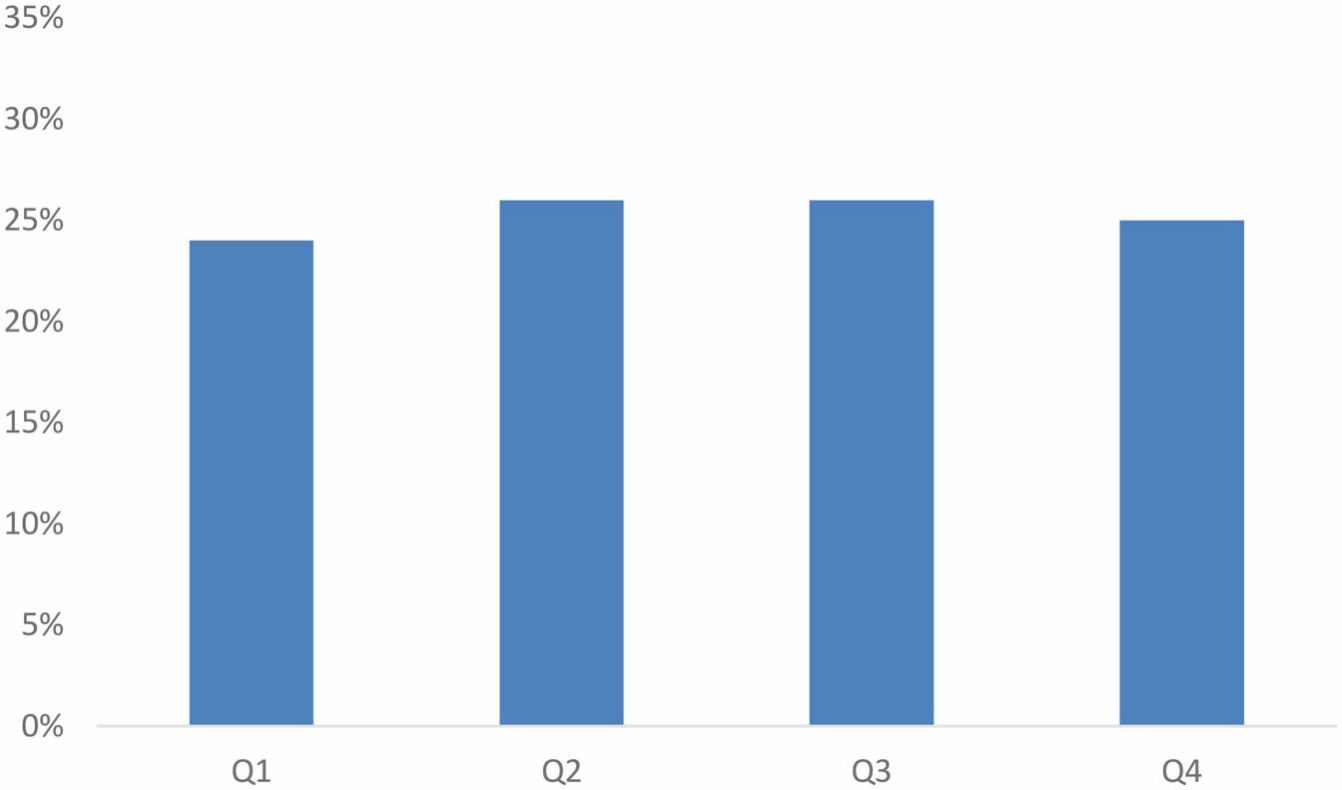
Distribution of players across the four quartiles.

A significant majority of the 15,734 players played first person shooter games (10,361), with multiplayer online battle arena games (3,691), real-time strategy games (561) and traditional sports games (571) making up a total of 4,823 players (the remaining 550 played more than one game. As can be seen in Figure 1, the distribution of birthdays across quartiles was quite uniform. A chi-square goodness of fit test indicated that there were statistically significant differences between the four quartiles [*χ*^2^ (3, *N* = 15,734) = 24.38, *p* < .001], however, the effect size was negligible (Cohen's w = .039). A binomial test of proportions indicated that Q1 is <25% (*p* < .001), Q2 (*p* = .182) and Q4 (*p* = .407) are equal to 25% and Q3 is >25% (*p* < .001).

The contingency tables (see Table 1) did not reveal any practically meaningful relative age effects in any of the age groupings (*χ*^2^ [6, *N* = 15,734] = 30.14, *p* < .001, Cramer's V = .044 or the various game types [*χ*^2^ (12, *N* = 15,734) = 28.64, *p* = .004, Cramer's V = .028; the benchmarks for small effect sizes for the respective analyses were .071 and .058, meaning that both effect sizes were negligible].

**Table 1:** Contingency tables for the age groups and type of games.

Category	*n*				
	Q1	Q2	Q3	Q4	Total
<24 years old	1,285	1,294	1,115	1,130	5,024
24–27 years old	1,122	1,315	1,327	1,295	5,059
>27 years old	1,303	1,397	1,488	1,463	5,651
More than one game	133	136	159	122	550
Multiplayer online battle arena	899	889	963	940	3,691
Real-time strategy	140	143	149	129	561
Traditional sports game	168	135	119	149	571
First person shooter game	2,370	2,703	2,740	2,548	10,361

## Discussion

The present study investigated whether relative age effects are present among professional esports athletes. Using a dataset of 15,734 professional athletes across ten major games sourced from Liquipedia, no practically meaningful differences in birth distributions were found. Although chi-square tests were statistically significant, all effect sizes were negligible (Cramer's V < .05). This suggests that, unlike many traditional sports (6, 7), professional esports do not exhibit systematic advantages for athletes born earlier in the selection year.

These results also diverge from Jakobsson et al. (24), who reported relative age effects among younger esports players and an inverse effect among adults in a heterogeneous national sample. A possible explanation lies in that relatively older youth may enjoy some temporary cognitive or experiential advantages, but these diminish as players progress. Because esports athletes typically train and compete online, independent of age-based groupings, younger players can develop at their own pace, unlike traditional sports where older players within the same cohort often enjoy physical and cognitive advantages. Moreover, esports rely more on cognitive abilities, reaction time, and strategic thinking (29, 30) than on physical maturity, which reduces the impact of small age differences. Consistent with evidence from other technical sports (12), relative age effects are less prevalent when success depends primarily on skill and practice rather than physical development.

Traditional sports often apply early selection into clubs, academies and federations, that may favour relatively older or more mature athletes (5). In esports, advancement is typically performance-based via ranked ladders and online tournaments, so players can remain active and progress in an apprenticeship model without passing early selection gates. This structure reduces early exclusion and likely lowers dropout among relatively younger players, aligning with relative age effect-reducing practices (22, 23, 31). This may also help to explain our null findings and the divergence from Jakobsson et al. (24). A further possibility is that the inverse effect reported among the oldest players by Jakobsson and colleagues may reflect relatively younger athletes leaving traditional sports due to relative age effects and opting for esports. However, even if overrepresented in amateur esports, they may not be more likely to reach the professional level. That said, as esports professionalise, pathways to the professional level may increasingly mirror traditional sports, including early scouting, try-outs, and selection, which could introduce relative age effects.

Despite the growing competitiveness of esports (1), relative age effects do not appear to influence who reaches the professional level. This finding suggests that expertise in esports may develop under fairer conditions than in traditional sports, where relative age effects can shape opportunities and career trajectories (31). Furthermore, the absence of differences across different game genres indicates that esports share structural features, such as skill-based ranking systems, online competition, and a reduced reliance on physical maturity, that distinguish them from traditional sports.

Beyond the structural characteristics of competition, the developmental and career pathways of esports athletes may further explain the absence of relative age effects. Unlike traditional sports, where early selection filters by clubs and federations often favour physically mature or relatively older youth (6, 31), esports pathways are typically more open and self-directed. Aspiring players often progress through online ladders, ranked systems, or collegiate leagues that emphasize demonstrable skill rather than physical maturity or coach selection (22). This openness may reduce early exclusion and allow players of all ages to advance based on performance alone, limiting the developmental pressures that produce relative age effects in traditional contexts. Esports careers also tend to peak and decline earlier than in most physical sports, with changes in cognitive and perceptual factors such as reaction time, attentional control, and decision-making efficiency being more relevant than physical deterioration (32).

The three mechanisms proposed by Hancock et al. (19) also help to interpret these findings. In traditional sports, the Matthew Effect, the Pygmalion Effect, and the Galatea Effect typically reinforce advantages for athletes born earlier in the year. In esports, however, the lack of age-based grouping weakens these processes, meaning that any advantages are more likely tied to technical skill rather than maturity. As a result, such mechanisms are less likely to produce relative age effects in esports.

Despite the strength of using a large dataset, this study has limitations. First, it focused exclusively on professional esports athletes. Relative age effects may still exist in youth or amateur esports populations, where developmental differences are more pronounced (5). Second, regional and cultural differences may play a role. In some contexts, children may face a choice between esports and traditional sports, and this could influence whether relative age effects emerge. Third, in line with the general trend in the literature [e.g., (8, 27, 28)], we chose a small effect size as a benchmark for practical meaningfulness. While a higher benchmark could have potentially impacted our conclusions, the effect sizes in this study were too low to ever be considered meaningful in this context (i.e., negligible). Finally, relying on Liquipedia as the sole data source may introduce biases, either because of incomplete coverage of professional players or inaccuracies in reported birthdates.

## Conclusion

The findings of this study indicate that professional esports do not exhibit practically meaningful relative age effects, setting them apart it from most findings from traditional sports. This lack of relative age effects can likely be attributed to the cognitive and skill-based nature of esports, as well as the flexible and online nature of competitive environments in this domain. However, esports have closely mirrored traditional sports when it comes to economic opportunities and prestige, which suggests that the issue of early talent identification could become more prominent in esports in the future. Early cognitive and technical skill assessments might therefore favour slightly older individuals within an age cohort, who may have had more time to develop these skills. Such subtle advantages could result in more opportunities and resources being allocated to these athletes, potentially perpetuating a selection bias similar to the relative age effect seen in traditional sports. If early talent identification in esports does emerge, future research could investigate potential relative age effects within such programs. In doing so, research and awareness about the phenomenon could increase the likelihood of esports remaining an accessible and equitable field for all aspiring athletes, regardless of their birthdate.

## Data Availability

The datasets presented in this study can be found in online repositories. The names of the repository/repositories and accession number(s) can be found below: https://doi.org/10.6084/m9.figshare.26129230.v1.

## References

[1] Ward MR Harmon AD. ESport superstars. J Sports Econom. (2019) 20(8):987–1013. 10.1177/1527002519859417

[2] Jackson RC Comber G. Hill on a mountaintop: a longitudinal and cross-sectional analysis of the relative age effect in competitive youth football. In: Williams AM, editor. Science and Football. New York, NY: Routledge (2023). p. 156–62. 10.1080/02640414.2019.1706830 31916503

[3] Barnsley RH Thompson AH Barnsley PE. Hockey success and birthdate: the relative age effect. Canad Assoc Health Phys Ed Recreat. (1985) 51(1):23–8.

[4] Lemoyne J Huard Pelletier V Trudeau F Grondin S. Relative age effect in Canadian hockey: prevalence, perceived competence and performance. Front Sports Act Living. (2021) 3:622590. 10.3389/fspor.2021.622590 33748753 PMC7969529

[5] Rees T Hardy L Güllich A Abernethy B Côté J Woodman T The great British medalists project: a review of current knowledge on the development of the world’s best sporting talent. Sports Med. (2016) 46(8):1041–58. 10.1007/s40279-016-0476-2 26842017 PMC4963454

[6] Yagüe JM de la Rubia A Sánchez-Molina J Maroto-Izquierdo S Molinero O. The relative age effect in the 10 best leagues of male professional football of the union of European football associations (UEFA). J Sports Sci Med. (2018) 17(3):409–16. PMID: 30116114 PMC6090398

[7] Yagüe JM Salguero A Villegas A Sánchez-Molina J Molinero O. The relative age effect in the two professional men’s football leagues in Spain. J Sports Sci Med. (2023) 22(4):700. 10.52082/jssm.2023.700 38045751 PMC10690514

[8] Smith KL Weir PL Till K Romann M Cobley S. Relative age effects across and within female sport contexts: a systematic review and meta-analysis. Sports Med. (2018) 48(6):1451–78. 10.1007/s40279-018-0890-8 29536262

[9] Pedersen AV Aune TK Dalen T Lorås H. Variations in the relative age effect with age and sex, and over time—elite-level data from international soccer world cups. PLoS One. (2022) 17(4):e0264813. 10.1371/journal.pone.0264813 35482636 PMC9049515

[10] Helsen WF Baker J Michiels S Schorer J Van Winckel J Williams AM. The relative age effect in European professional soccer: did ten years of research make any difference? J Sports Sci. (2012) 30(15):1665–71. 10.1080/02640414.2012.721929 23005576

[11] Bjerke Ø Lorås H Pedersen AV. Variations of the relative age effect within and across groups in elite alpine skiing. Comprehens Psychol. (2016) 5:2165222816648077. 10.1177/2165222816648077

[12] van Rossum JH. Relative age effect revisited: findings from the dance domain. Percept Mot Skills. (2006) 102(2):302–8. 10.2466/pms.102.2.302-308 16826648

[13] Breznik K Law KM. Relative age effect in mind games: the evidence from elite chess. Percept Mot Skills. (2016) 122(2):583–94. 10.1177/0031512516640957 27166336

[14] Helsen WF Baker J Schorer J Steingroever C Wattie N Starkes JL. Relative age effects in a cognitive task: a case study of youth chess. High Ability Stud. (2016) 27(2):211–21. 10.1080/13598139.2016.1242063

[15] Patalay P Belsky J Fonagy P Vostanis P Humphrey N Deighton J The extent and specificity of relative age effects on mental health and functioning in early adolescence. J Adolesc Health. (2015) 57(5):475–81. 10.1016/j.jadohealth.2015.07.012 26385065

[16] Frisira E Holland J Sayal K. Systematic review and meta-analysis: relative age in attention-deficit/hyperactivity disorder and autism spectrum disorder. Eur Child Adolesc Psychiatry. (2024) 34:381–401. 10.1007/s00787-024-02459-x 38767699 PMC11868292

[17] Arnold G Depew B. School starting age and long-run health in the United States. Health Econ. (2018) 27(12):1904–20. 10.1002/hec.3810 30073733

[18] Dobkin C Ferreira F. Do school entry laws affect educational attainment and labor market outcomes? Econ Educ Rev. (2010) 29(1):40–54. 10.1016/j.econedurev.2009.04.003

[19] Hancock DJ Adler AL Côté J. A proposed theoretical model to explain relative age effects in sport. Eur J Sport Sci. (2013) 13(6):630–7. 10.1080/17461391.2013.775352 24251740

[20] Hurley WJ. Equitable birthdate categorization systems for organized minor sports competition. Eur J Oper Res. (2009) 192(1):253–64. 10.1016/j.ejor.2007.09.005

[21] Mann DL van Ginneken PJ. Age-ordered shirt numbering reduces the selection bias associated with the relative age effect. J Sports Sci. (2017) 35(8):784–90. 10.1080/02640414.2016.1189588 27238077

[22] Fisackerly W Hwang Y. Where do amateurs go to become pros? A comparison of the current competition systems in collegiate esports to traditional collegiate sport environments. J Electron Gaming Esports. (2024) 2(1):jege.2023-0009. 10.1123/jege.2023-0009

[23] Zhao Y Meng-Lewis Y Ni B Lewis G Lin Z. The continuity of eSports athletes’ careers: skill transformation, personal development, and well-being. Sage Open. (2024) 14(1):1–13. 10.1177/21582440241249263

[24] Jakobsson J Julin AL Persson G Malm C. Darwinian selection discriminates young athletes: the relative age effect in relation to sporting performance. Sports Med Open. (2021) 7:1–18. 10.1186/s40798-021-00300-2 33650038 PMC7921243

[25] Liquipedia. Notability Guidelines for Counter Strike (n.d.). Available online at: https://liquipedia.net/counterstrike/Liquipedia:Notability_Guidelines (Accessed August 7, 2024)

[26] Liquipedia. Notability Guidelines for Valorant (n.d.). Available online at: https://liquipedia.net/valorant/Liquipedia:Notability_Guidelines (Accessed August 7, 2024)

[27] Cobley S Baker J Wattie N McKenna J. Annual age-grouping and athlete development: a meta-analytical review of relative age effects in sport. Sports Med. (2009) 39:235–56. 10.2165/00007256-200939030-00005 19290678

[28] Bozděch M Agricola A Zháněl J. The relative age effect at different age periods in soccer: a meta-analysis. Percept Mot Skills. (2023) 130(6):2632–62. 10.1177/00315125231210585 37903410

[29] Larsen LJ. The play of champions: toward a theory of skill in eSport. Sport Ethics Phil. (2022) 16(1):130–52. 10.1080/17511321.2020.1827453

[30] Toth AJ Ramsbottom N Constantin C Milliet A Campbell MJ. The effect of expertise, training and neurostimulation on sensory-motor skill in esports. Comput Human Behav. (2021) 121:106782. 10.1016/j.chb.2021.106782

[31] Webdale K Baker J Schorer J Wattie N. Solving sport’s ‘relative age’ problem: a systematic review of proposed solutions. Int Rev Sport Exerc Psychol. (2020) 13(1):187–204. 10.1080/1750984X.2019.1675083

[32] Lin Z Zhao Y. Self-enterprising eSports: meritocracy, precarity, and disposability of eSports players in China. Int J Cult Stud. (2020) 23(4):582–99. 10.1177/1367877920903437

